# Survival status and predictors of mortality among asphyxiated neonates admitted to the NICU of Dessie comprehensive specialized hospital, Amhara region, Northeast Ethiopia

**DOI:** 10.1371/journal.pone.0279451

**Published:** 2022-12-21

**Authors:** Yibeltal Asmamaw Yitayew, Zemen Mengesha Yalew

**Affiliations:** 1 Department of Pediatrics Nursing, College of Medicine and Health Science, Wollo University, Dessie, Amhara Region, Ethiopia; 2 Department of Comprehensive Nursing, College of Medicine and Health Science, Wollo University, Dessie, Amhara Region, Ethiopia; WCU: Wachemo University, ETHIOPIA

## Abstract

**Introduction:**

Birth asphyxia is one of the leading causes of early neonatal mortality, which causes an estimated 900,000 deaths annually. Therefore, assessing the survival status and predictors of mortality among asphyxiated neonates will be highly helpful to policymakers in designing, implementing, and evaluating programs to achieve the sustainable development goal of reducing neonatal mortality as low as 12/1,000 live births by 2030.

**Methods:**

A facility-based retrospective cohort study was conducted among 378 asphyxiated neonates admitted to the NICU of Dessie Comprehensive Specialized Hospital from January, 2017 –December, 2019. The data were collected from eligible records by using a structured data extraction tool from March 30 –April 21, 2020. The data were cleaned manually and entered into Epi-data version 7.1.2.0, and STATA version 16 was used for the analysis. Bivariate and Multivariate Cox proportional hazard regression analysis were performed, and significant predictors were identified using 95% confidence interval and p-value <0.05.

**Result:**

A total of 378 neonates were followed for 2298 neonatal days, ranging from 1 to 28 days. The mortality incidence rate was 5.3/100 person-days-of observation (95% CI: 4.41, 6.29), and 32% (95% CI: 27.6%, 36.8%) of the study subjects died. Admission weight (AHR: 1.72; 95% CI: 1.09, 2.72), seizure (AHR: 1.52; 95% CI: 1.02, 2.27), neonates who received resuscitation (AHR: 2.11; 95% CI: 1.18, 3.80), and stage of asphyxia (moderate (AHR: 3.50; 95% CI: 1.55, 8.36), and severe (AHR: 11.55; 95% CI: 4.73, 28.25)) were significant predictors of neonatal mortality among asphyxiated neonates.

**Conclusion:**

The magnitude of neonatal mortality among asphyxiated neonates in the study area was high. Admission weight, seizure, resuscitation, and stage of asphyxia were significant predictors of mortality among neonates with asphyxia. Therefore, special attention should be given to asphyxiated neonates with low admission weight and those who had seizure. Additionally, the timing, quality, and effectiveness of resuscitation might need further assessment and evaluation.

## Introduction

The neonatal period is the most vulnerable time for a child’s survival, with an average of 18 deaths per 1,000 live births globally in 2017 [1]. The child’s risk of dying is highest in the first 28 days of life. Globally, 2.5 million children died in the first months of life in 2017, which accounts for 47% of all under-five child deaths. Preterm birth, birth asphyxia, infection, and birth defects cause most neonatal deaths [2]. Asphyxia is defined as a lack of gas exchange that causes elevation in carbon dioxide levels, and resulting in a combined respiratory and metabolic acidosis [3]. Birth asphyxia can be classified as mild, moderate, or severe based on the appearance, pulse, grimace, activity and respiration (APGAR) score in the first, fifth, and tenth minutes of life [4].

Birth asphyxia is one of the leading causes of early neonatal mortality, and accounts for an estimated 900,000 deaths worldwide each year [3, 5]. It is estimated that birth asphyxia accounts for about 23% of all newborn deaths worldwide [6]. The incidence of birth asphyxia has decreased significantly in most industrialized countries and accounts for less than 0.1% of newborn infant deaths. However, in developing countries, the rate of birth asphyxia is much higher, and the case fatality rate is ≥ 40% [7].

Birth asphyxia is the leading cause of neonatal mortality in low and middle-income countries, and it is also the primary cause of long-term neurodevelopmental disorders [8]. In East Africa, the neonatal mortality rate ranges from 11–102 per 1000 live births, and birth asphyxia was the primary cause of neonatal death in 2015 (31.6%) [6]. In Ethiopia, birth asphyxia is the second leading cause of neonatal mortality (26.7%) and the fourth most common cause of death for children under the age of five (11.3%) [9].

Birth asphyxia has a wide range of complications and can affect the motor, sensory, cognitive, psychological development of the newborn. Even though the majority of infants with perinatal asphyxia recover quickly, a small percentage may suffer from an evolving clinical encephalopathy (hypoxic-ischemic encephalopathy). Approximately 20–30% of infants with hypoxic-ischemic encephalopathy die in the neonatal period, and 33–50% of survivors have permanent neurodevelopmental abnormalities (cerebral palsy, mental retardation) [10, 11].

There are several risk factors for mortality in newborns with asphyxia. Prematurity, maternal fever, multiple births, maternal anemia, neonates requiring resuscitation, neonates presenting with convulsions or cyanosis, prolonged capillary refill, hypoxic ischemic-encephalopathy stage III, low APGAR score (0–3), coma, prolonged seizures refractory to therapy, fetal distress, end-organ dysfunction and congenital brain malformation are some of the risk factors [12–14]. Studies conducted in Ethiopia also revealed that neonatal sepsis, preterm birth, antepartum hemorrhage, postpartum hemorrhage, cord prolapse, pregnancy induced hypertension, maternal iron deficiency anemia, low birth weight, stage II and III hypoxic ischemic encephalopathy, seizure, thrombocytopenia were significant predictors of mortality among asphyxiated neonates [15–17].

In Ethiopia, birth asphyxia is the leading cause of neonatal mortality [18]. Thus, identifying predictors of mortality will be highly useful to program managers and policymakers in designing, implementing, and evaluating programs to achieve the sustainable development goal of reducing neonatal mortality as low as 12/1,000 live births by 2030 [19]. Therefore, the purpose of this study was to assess the survival status and predictors of mortality among asphyxiated neonates admitted to the NICU of Dessie Comprehensive Specialized Hospital (DCSH).

## Materials and methods

### Study area and setting

The study was conducted in Dessie Comprehensive Specialized Hospital (DCSH), which is located in Dessie town, the capital city of the South Wollo zone. Dessie town is located 401km away from Addis Ababa, the capital city of Ethiopia, and 480 km away from Bahir Dar, the capital city of the Amhara regional state. According to the 2019 central statistics agency of Ethiopia population projection report, Dessie town had 268,931 population [20]. There are two public hospitals (DCSH and Borumeda hospital) and four public health centers (Dessie health center, Segno Gebaya health center, Buanbuawha health center, and Kurkur health center) in Dessie town. During the study period, DCSH had 1140 NICU admissions each year on average, with asphyxia, prematurity and congenital anomalies being the most common reasons for admission.

### Study design and period

A facility-based retrospective cohort study was conducted among asphyxiated neonates admitted to the NICU of Dessie Comprehensive Specialized Hospital from January, 2017 –December, 2019. The data collection was conducted from March 30 –April 21, 2020.

### Source population

All asphyxiated neonates admitted to the NICU of Dessie Comprehensive Specialized Hospital, Dessie town, Ethiopia.

### Study population

All eligible asphyxiated neonates admitted to the NICU of Dessie Comprehensive Specialized Hospital from January, 2017 –December, 2019.

### Eligibility criteria

#### Exclusion criteria

Asphyxiated neonates with unknown admission or discharge date.

### Sample size determination and sampling procedure

The study subjects were selected from the NICU admission registration logbook. The registration logbook contains data such as admission date, age, medical diagnosis, medical registration number (MRN), etc. From January 2017—December 2019, 454 asphyxia cases were registered in the NICU admission logbook. The medical records of asphyxiated neonates were obtained from the card room using their MRN numbers. Among the 454 asphyxia cases, 8 cards were lost, 32 cards had a different diagnosis, and 36 cards had significant missing data. Finally, 378 cards were included in the final analysis.

### Operational definition/Definition of terms

#### Survival status

Survival status was determined based on the health condition of the asphyxiated newborn at the time of discharge from the NICU (death, recovery, referral or discharge against medical advice).

#### Asphyxiated neonate

Newborns admitted to the NICU for the medical diagnosis of asphyxia.

#### Comorbidity

Any other underling health problem in the newborns in addition to asphyxia (meconium aspiration syndrome, sepsis, jaundice, etc.).

#### Obstetric complication

Any of the following: Gestational diabetics, preeclampsia, antepartum haemorrhage, intrapartum fever, premature placental separation.

#### Labor and delivery complication

Any of the following: Abnormal presentations, cephalopelvic disproportion, cord prolapse, preterm labor.

#### Stages of asphyxia

For this study, the stage of asphyxia was taken from the medical record based on the physicians’ diagnosis. However, based on the APGAR score, asphyxia can be classified as mild or stage I (6–7), moderate or stage II (4–5) and severe or stage III (0–3) [21].

### Data collection tools and techniques

The data were collected using a semi-structured data extraction tool. Five neonatal nurses collected the data, and one MSc in pediatric nursing professional supervised the process. The MRN of asphyxiated neonates admitted to the NICU of Dessie Comprehensive Specialized Hospital from January, 2017- December, 2019 was obtained from the NICU admission registration logbook, and the cards of study subjects were obtained from the card room using their MRN numbers.

### Data quality assurance

The quality of the data was ensured through training of data collectors, regular supervision, immediate feedback, and spot-checking. Moreover, the supervisors and principal investigators daily checked the completeness of the collected data. Pretesting of the data extraction tool was undertaken on 5% (23) of the sample size. The goodness of fit test for the Cox-proportional hazard regression model was tested using Cox-Snell residuals, and the Cox model assumptions were tested using Schoenfeld residuals (Global test, P-value = 0.152).

### Data processing and analysis

All field questionnaires were checked for completeness, consistency, and accuracy. Then the data were entered into EPI data (version 7.1.2.0) and exported to STATA version 16 for analysis. Descriptive statistics (frequency table, pie chart, and bar graph) were used to summarize the data. The Kaplan-Meier and Cox regression were used to assess the association of the independent variables with the outcome. Variables with a p-value ≤0.2 in bivariate Cox regression were further analyzed using multivariate cox regression. The adjusted hazard ratio (AHR) with 95% confidence interval (CI) from the multivariate cox regression was used as a measure of association, and variables with a p-value <0.05 were considered significant predictors.

### Ethics approval and consent to participate

Ethical clearance and approval were obtained from Wollo University, College of Medicine and Health Sciences (RF: 163/02/12). Official letters were written to DSCH, and permission to conduct the study was obtained from the responsible authorities of the hospital. Since this is a retrospective study, getting permission from study subjects was not possible. Therefore, consent to review medical records was waived by the authorities of DSCH. The information obtained was kept confidential through anonymous recording and coding of the data extraction tool, and compliance was ensured in accordance with the Declaration of Helsinki.

## Result

### Socio-demographic factors of the study subjects

The majority of mothers (57.9%) were aged 25–34 years (mean ± SD = 26.6±4.7 years), and 58.2% lived in rural areas. The majority of neonates (61.4%) were males, and 63.5% were aged 1–24 hours at the time of NICU admission (Table 1).

**Table 1:** Sociodemographic characteristics of the study subjects in DCSH, Amhara region, Northeast Ethiopia (N = 378).

Variable	Categories	Frequency (N)	Percent (%)
Age of the mother	< 25 years	126	33.3
	25–34 years	219	57.9
	≥ 35 years	33	8.7
Residence	Urban	158	41.8
	Rural	220	58.2
Sex of the newborn	Male	232	61.4
	Female	146	38.6
Age of the newborn at admission	< 1 Hr	106	28.0
	1–24 Hr	240	63.5
	>24 Hr	32	8.5

### Reproductive health related characteristics of the mothers

The majority of mothers (92.6%) attended ANC, 59.8% were primiparous, 92.1% had no obstetric complications, and 33.3% had delivery complications. The vast majority of mothers (95.2%) gave birth in the health facilities, 64.3% had spontaneous vaginal delivery (SVD), 36.2% had prolonged labor, 12.4% had PROM, and 2.1% had multiple deliveries (Table 2).

**Table 2:** Reproductive health related characteristics of the mothers in DCSH, Amhara region, Northeast Ethiopia (N = 378).

Variable	Categories	Frequency (N)	Percent (%)
ANC visit	Yes	350	92.6
	No	28	7.4
Obstetric complications	Yes	30	7.9
	No	348	92.1
Parity	Primigravida	226	59.8
	Multigravida	152	40.2
Place of delivery	Health facility	360	95.2
	Home	18	4.8
Mode of delivery	SVD	243	64.3
	Assisted VD	67	17.7
	C/S	68	18
Duration of labor	Prolonged	137	36.2
	Normal	241	63.8
Was there PROM	Yes	47	12.4
	No	331	87.6
Delivery complication	Yes	126	33.3
	No	252	66.7
Multiple birth	Yes	8	2.1
	No	370	97.9

### Newborn health related characteristics

The majority (85.7%) of the neonates weighed ≥2500 g at admission (mean ± SD, 2.85±0.47 g), and 85.7% were term. The majority (54.8%) of newborns had a first-minute APGAR score of 4–5, 79.4% received resuscitation, 69.3% had hypothermia, 22.5% had comorbidity, 62.2% had stage II asphyxia, and 24.6% had seizures (Table 3).

**Table 3:** Newborn health related characteristics of the study subjects in DCSH, Amhara region, Northeast Ethiopia (N = 378).

Variable	Categories	Frequency (N)	Percent (%)
Weight at admission	< 2500 g	54	14.3
	≥ 2500 g	324	85.7
Gestational age	Preterm	17	4.5
	Term	324	85.7
	Unknown	37	9.8
First minute APGAR score	≤ 3	61	16.1
	4–5	207	54.8
	6–7	85	22.5
	8–10	2	0.5
	Unknown	23	6.1
Fifth minute APGAR score	≤ 3	8	2.1
	4–5	120	31.7
	6–7	178	47.1
	8–10	42	11.1
	Unknown	30	7.9
Required resuscitation	Yes	300	79.4
	No	78	20.6
Hypothermia	Yes	262	69.3
	No	116	30.7
Comorbidity	Yes	293	77.5
	No	85	22.5
Stage of Asphyxia	Mild	90	23.8
	Moderate	235	62.2
	Severe	53	14
Was there seizure	Yes	93	24.6
	No	285	75.4

### Survival status

**Fig 1:**
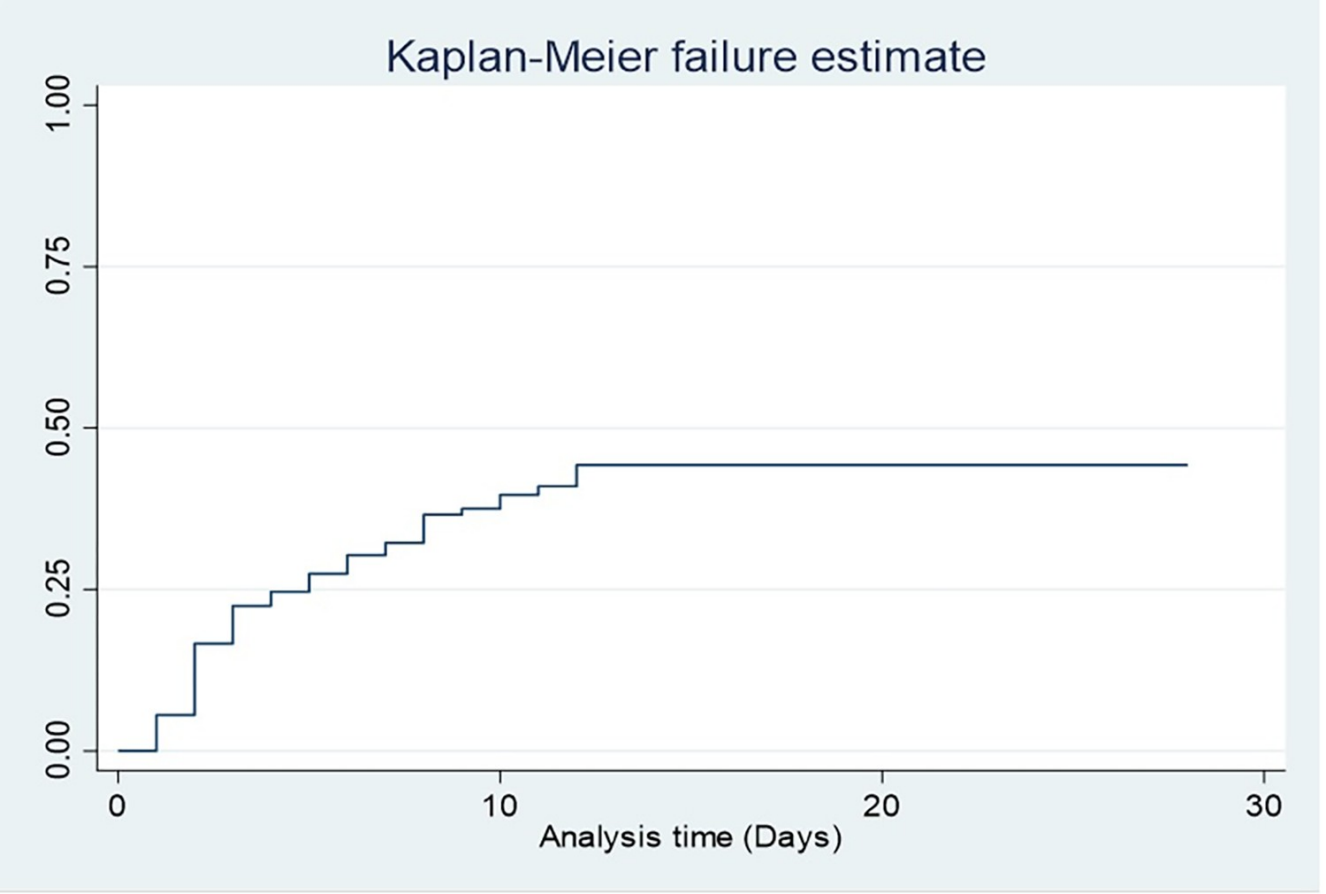
Kaplan-Meier failure estimate of asphyxiated newborns admitted to the NICU of DCSH, Amhara region, Northeast Ethiopia (N = 378).

**Fig 2:**
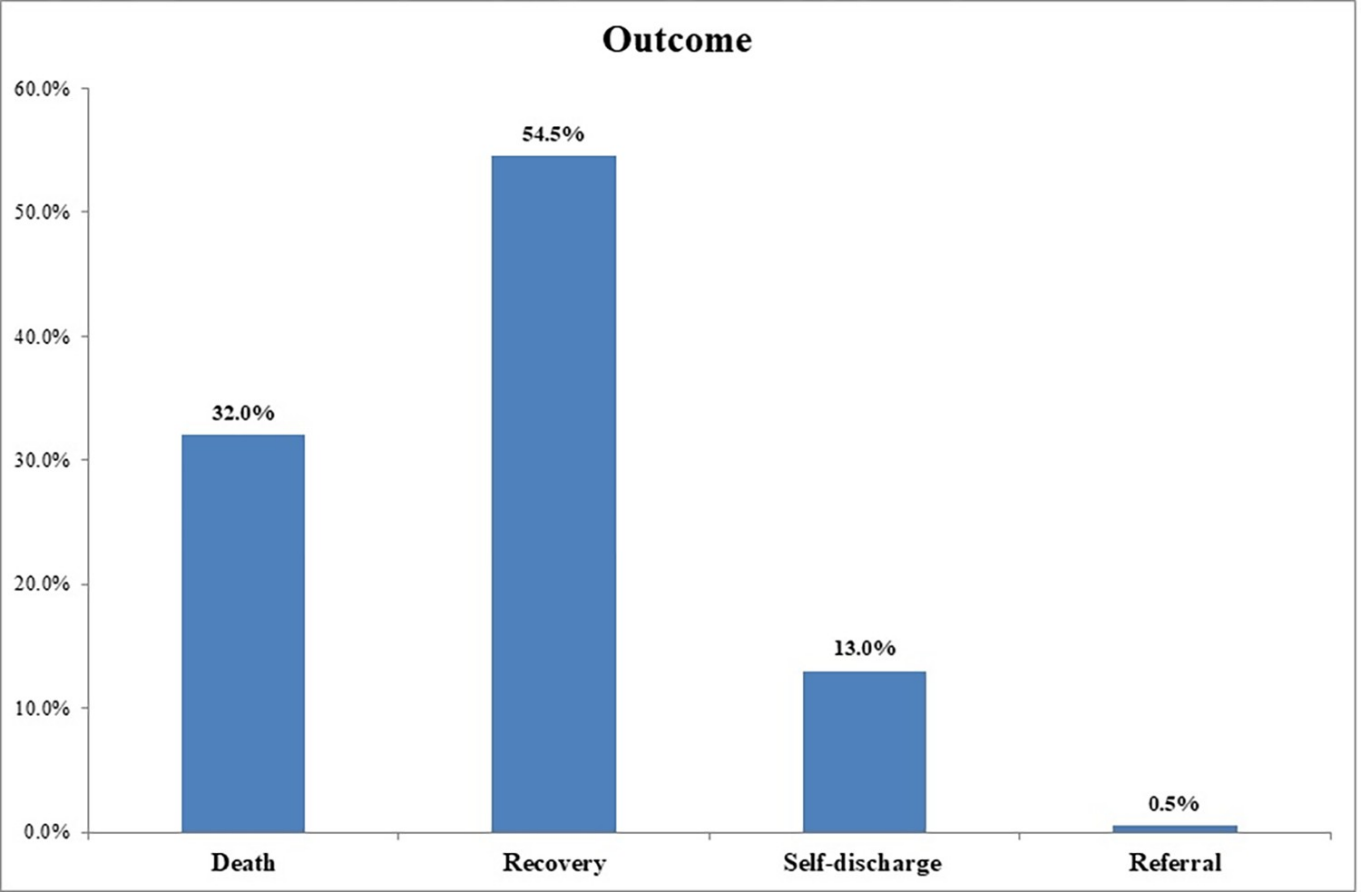
The outcome of asphyxiated newborns admitted to the NICU of DCSH, Amhara region, Northeast Ethiopia (N = 378).

**Fig 3:**
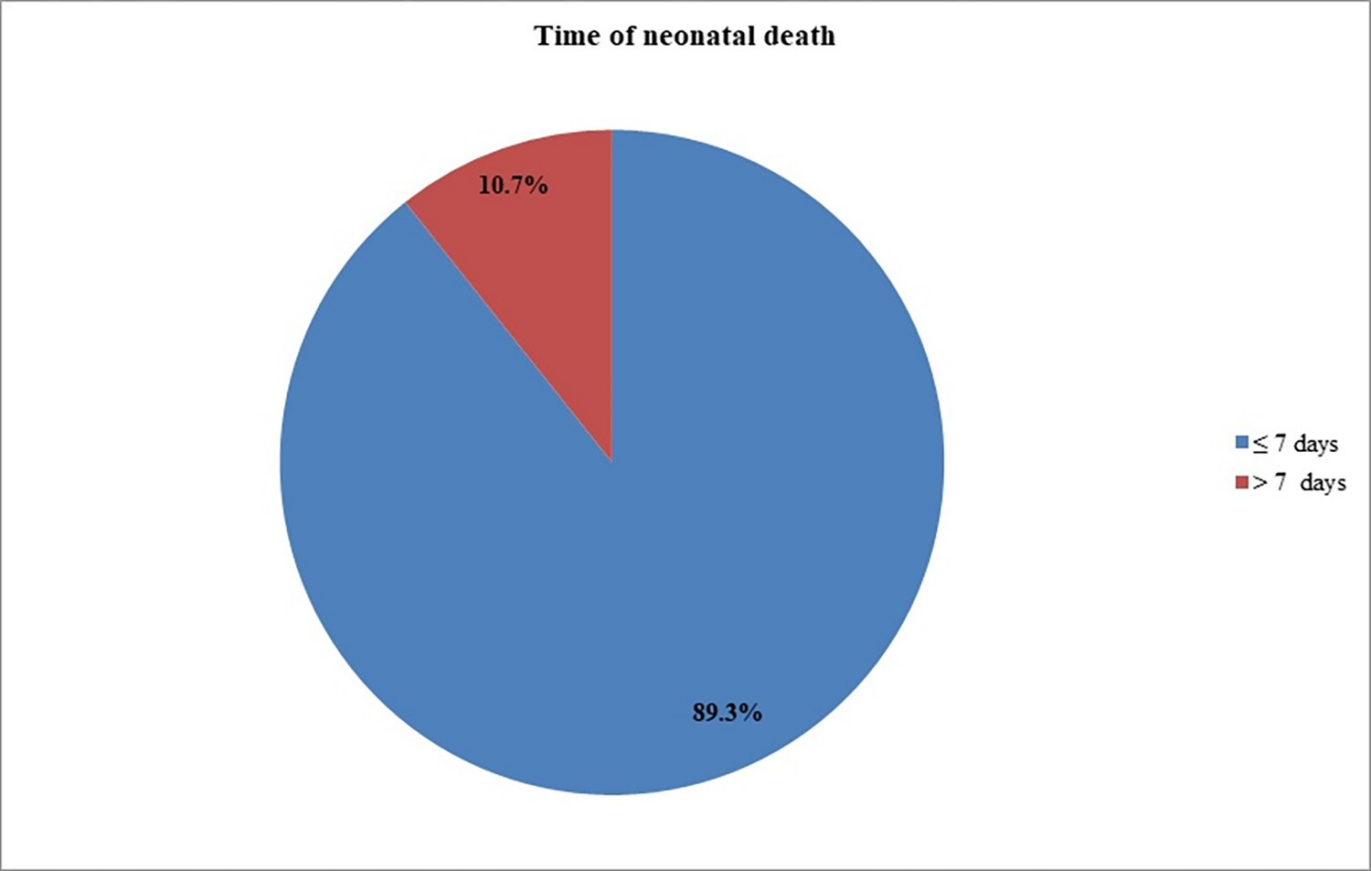
The occurrence of neonatal death among asphyxiated newborns admitted to the NICU of DCSH, Amhara region, Northeast Ethiopia (N = 378).

**Fig 4:**
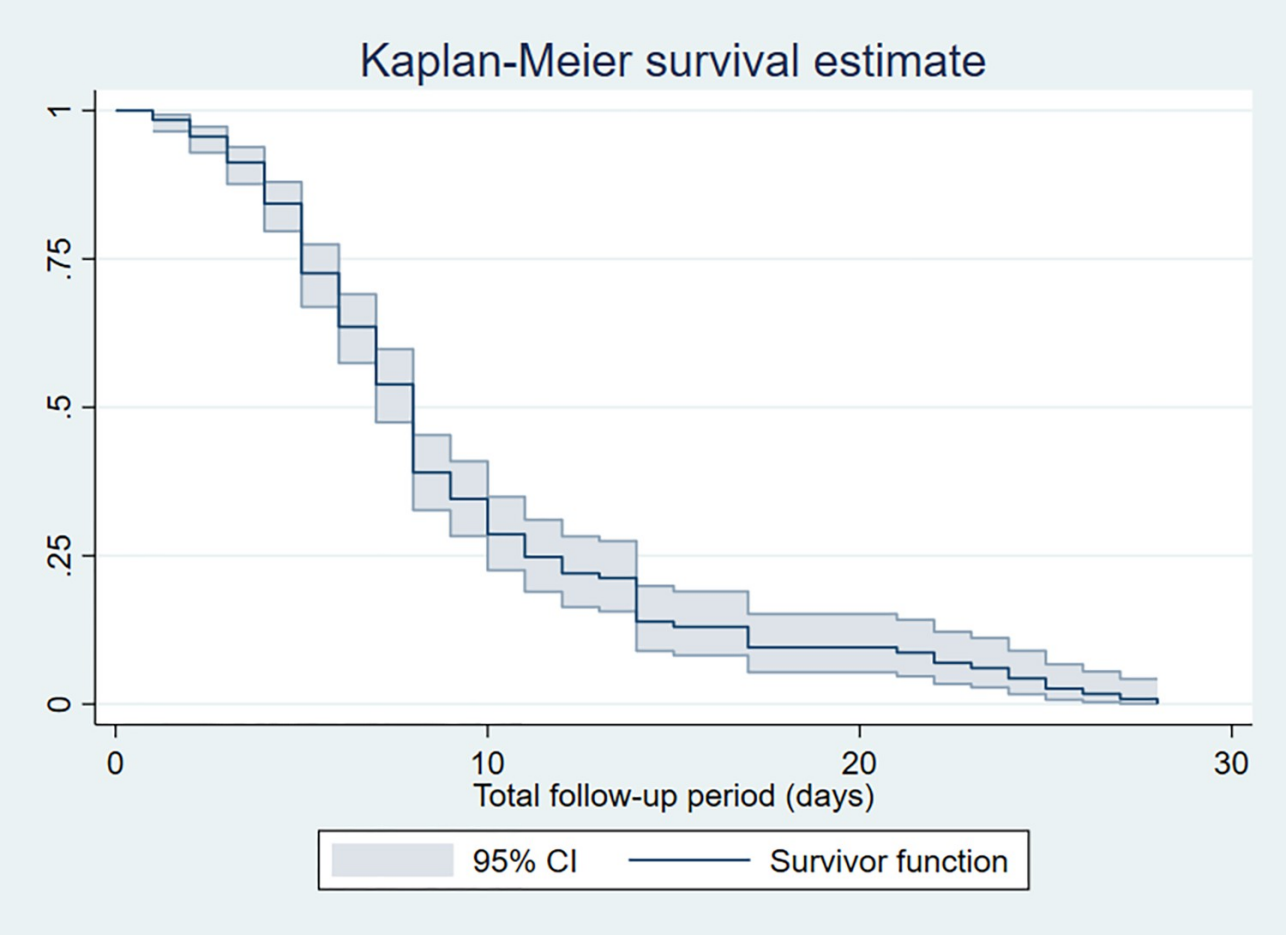
Kaplan-Meier survival estimate of asphyxiated newborns admitted to the NICU of DCSH, Amhara region, Northeast Ethiopia (N = 378).

A total of 378 neonates were followed for 2298 neonatal days with a minimum of 1 day and a maximum of 28 days. Of the study subjects, 32% (95% CI: 27.6%, 36.8%) died, which resulted in a mortality incidence of 5.3/100 person-days-of observation (95% CI: 4.41, 6.29) **(Figs 1 and 2).** The vast majority (89.3%) of deaths occurred within one week of admission **(Fig 3).** On the other hand, 54.5% (95% CI: 49.5%, 59.5%) of study subjects recovered with a median survival time of 8 days (95% CI: 7.48, 8.5) **(Fig 4).**

### Kaplan-Meir failure analysis

**Fig 5:**
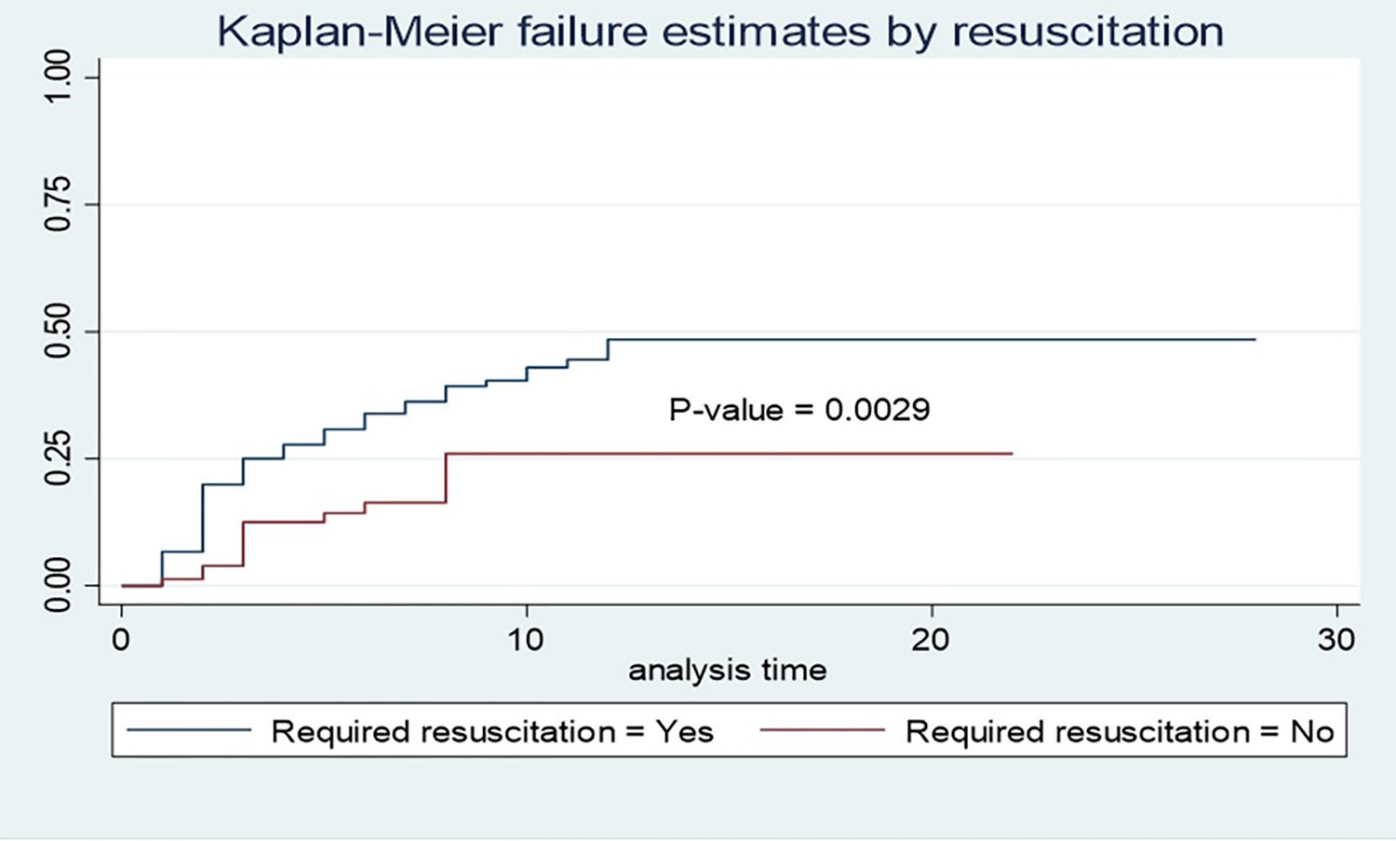
Kaplan-Meier failure curves to compare the mortality pattern of asphyxiated neonates based on resuscitation requirement in DCSH, Amhara region, Northeast Ethiopia (N = 378).

**Fig 6:**
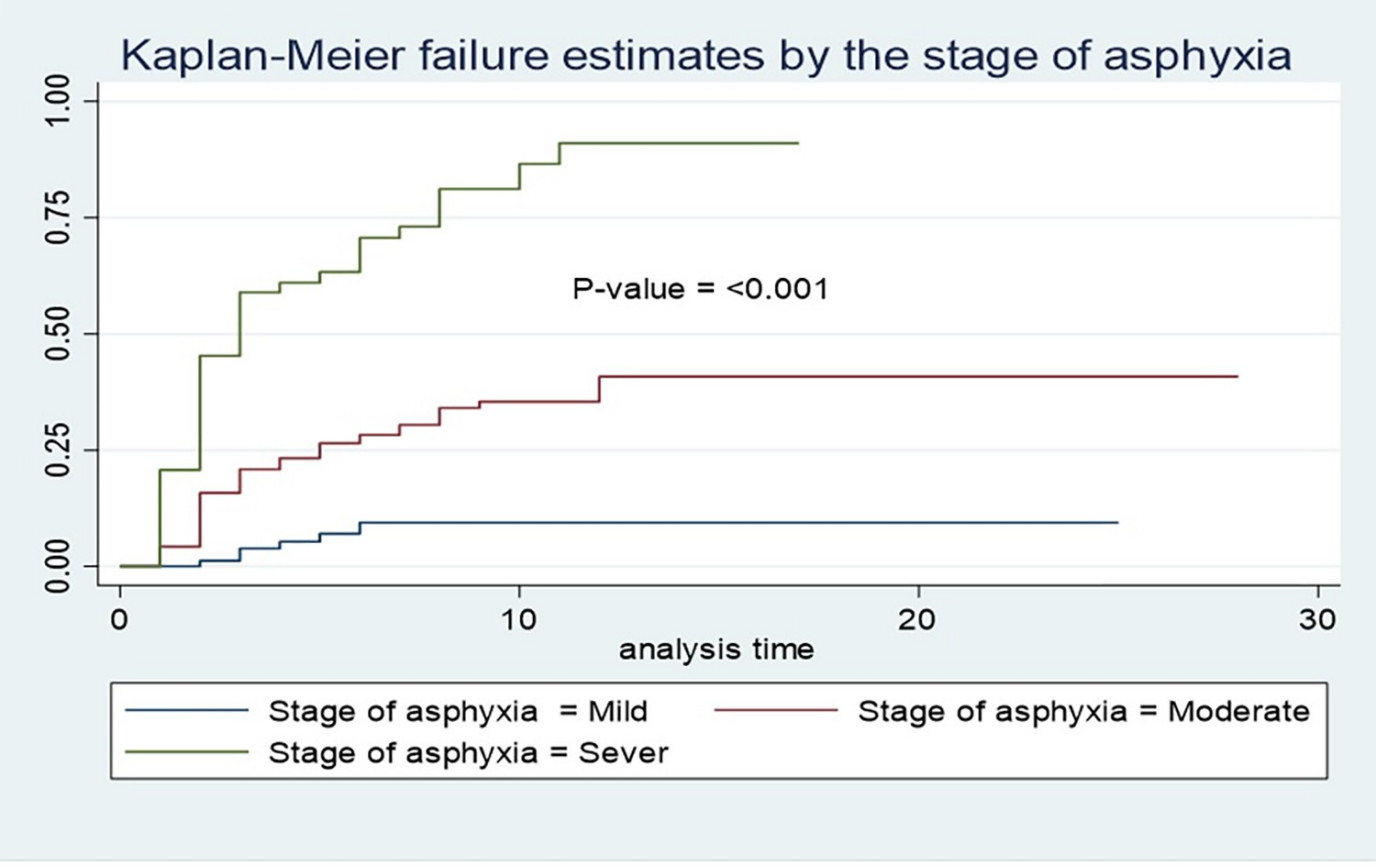
Kaplan-Meier failure curves to compare the mortality pattern of asphyxiated neonates based on the stage of asphyxia in DCSH, Amhara region, Northeast Ethiopia (N = 378).

STATA version 16 was used to analyze the log-rank test and the Kaplan-Meier failure curve. According to the findings, the mortality pattern of asphyxiated neonates varied across most of the covariates (Table 4). Residence, mode of delivery, duration of labor, delivery complications, admission weight, seizure, neonates who received resuscitation, and stage of asphyxia were variables that had a high effect on death among neonates with asphyxia **(Figs 5 and 6).**

**Table 4:** Log-rank estimate of variables on mortality of asphyxiated neonates admitted to the NICU of DCSH, Amhara region, Northeast Ethiopia (N = 378).

Variables	Log rank test	
	X^2^	P-value
Age of the mother	3.79	0.15
Residence	6.94	0.0084
Parity	2.63	0.105
Mode of delivery	8.82	0.0121
Duration of labor	4.37	0.0366
PROM	2.87	0.0904
Delivery complication	6.4	0.0114
Admission weight	5.4	0.0201
Seizure	28.6	<0.001
Required resuscitation	8.88	0.0029
Stage of asphyxia	71.31	<0.001

### Predictors of mortality among asphyxiated neonates

The bivariate Cox proportional hazard regression analysis showed a p-value of ≤0.2 for the following variables: age of the mother, residence, parity, mode of delivery, duration of labor, PROM, delivery complications, admission weight, seizure, requiring resuscitation, and stage of asphyxia. Further analysis of the above variables in the multivariable Cox proportional hazard regression revealed that admission weight, seizure, requiring resuscitation, and stage of asphyxia were significant predictors of neonatal mortality among asphyxiated neonates. The Cox-proportional hazard regression model assumptions were tested using the Cox-Snell residual and Schoenfeld residuals (Global test, P-value = 0.152).

Asphyxiated neonates weighing <2500g at admission had a 1.72 times higher risk of death than their counterparts (AHR: 1.72; 95% CI: 1.09, 2.72). Likewise, the risk of death was 1.52 times higher among asphyxiated newborns with seizures compared to those without seizures (AHR: 1.52; 95% CI: 1.02, 2.27). Additionally, asphyxiated neonates who received resuscitation had a 2.11 higher risk of death compared to their counterparts (AHR: 2.11; 95% CI: 1.18, 3.80). Finally, compared to neonates with mild asphyxia, neonates with moderate and severe asphyxia had a 3.50 (AHR: 3.50; 95% CI: 1.55, 8.36) and 11.55 (AHR: 11.55; 95% CI: 4.73, 28.25) times higher risk of mortality, respectively (Table 5).

**Table 5:** Predictors of mortality among asphyxiated neonates admitted to the NICU of DCSH, Amhara region, Northeast Ethiopia (N = 378).

Variables	Category	Survival status of asphyxiated neonates		Crude hazard ratio (95%CI)	AHR(95%CI)	P- value
		Censored	Died			
Age of the mother	< 25 years	87 (69%)	39(31%)	1.68 (0.71–3.96)	1.19 (0.47–2.98)	0.715
	25–34 years	143 (65.9%)	76 (34.1%)	2.03 (0.88–4.66)	1.57 (0.66–3.75)	0.306
	≥ 35 years	27 (81.8%)	6 (18.2%)	1	1	
Residence	Urban	120(75.9%)	38(24.1%)	1	1	
	Rural	137(62.3%)	83(37.7%)	1.65 (1.13–2.43)	1.45 (0.98–2.16)	0.064
Parity	Primigravida	146 (64.6%)	80 (35.4%)	1.36 (0.93–1.98)	1.37 (0.89–2.11)	0.156
	Multigravida	111(73%)	41 (27%)	1		
Mode of delivery	SVD	178 (40%)	65 (27.1%)	1		
	Assisted VD	39 (8.3%)	28 (11%)	1.87 (1.20–2.91)	1.06 (0.64–1.74)	0.826
	C/S	40 (2.4%)	28 (2.3%)	1.58 (1.01–2.46)	1.01 (0.56–1.81)	0.99
Duration of labor	Prolonged	83 (60.6%)	54 (39.4%)	1.47 (1.03–2.1)	1.00 (0.63–1.55)	0.97
	Normal	174 (72.2%)	67 (27.8%)	1		
Is there PROM	Yes	27 (57.4%)	20 (42.6)	1.55 (0.96–2.50)	1.63 (0.96–2.73)	0.071
	No	230 (69.5%)	101 (30.5%)	1		
Was there delivery complication	Yes	76 (60.3%)	50 (39.7%)	1.61 (1.12–2.31)	1.23 (0.77–1.97)	0.39
	No	181 (71.8%)	71 (28.2%)	1		
Weight at admission	<2500g	27 (50%)	27 (50%)	1.71 (1.11–2.62)	1.72 (1.09–2.72)*	0.02
	≥2500g	230 (71%)	94 (29%)	1		
Was there seizure	Yes	37 (39.8%)	56 (60.2%)	2.74 (1.92–3.92)	1.52 (1.02–2.27)*	0.038
	No	220 (71.2%)	65(22.8%)	1		
Requiring resuscitation	Yes	193 (64.3%)	107 (35.7%)	2.16 (1.24–3.77)	2.11 (1.18–3.80)*	0.012
	No	64 (82.1%)	14 (17.9%)	1		
Stage of asphyxia	Stage I	84 (93.3%)	6 (6.7%)	1		
	Stage II	163 (69.4%)	72 (30.6)	4.48 (1.95–10.3)	3.50 (1.55–8.36)*	0.003
	Stage III	10 (18.9%)	43 (81.1%)	16.4 (6.96–38.5)	11.55 (4.73–28.25)*	< 0.001

* Significant predictors at 95% CI

## Discussion

According to the findings of this study, 32% (95% CI: 27.5%-36.9%) of asphyxiated newborns admitted to the NICU of DSCH died, resulting in a mortality incidence rate of 5.3/100 person-days-of observation. Comparable findings were reported from studies conducted in Northwest Ethiopia (31.09%) [17] and rural Nigeria (31.1%) [22]. This finding is higher than studies conducted in Addis Ababa Ethiopia (24.09%) [15], Southern Ethiopia (7.85%) [16], Nigeria ((23.9%), (18%) and (20.8%)) [23–25], Tanzania (23%) [26], South Africa (13.3%) [27] and India ((20.24%) and (20%)) [28, 29]. However, higher neonatal mortality rate was reported from studies in Ethiopia (40%) [30], India (40.6%) [31], and Tanzania (62.5%) [32]. These discrepancies might be due to variations in hospital settings and resources, advances in hospital caring capacity, management protocols, and study design.

Admission weight of <2500g was a significant predictor of newborn mortality among asphyxiated neonates (AHR = 1.72). This could be because low birth weight neonates are more likely to be born prematurely and lack adequate surfactants, resulting in breathing difficulties, problems with cardiopulmonary transition, and, eventually, birth asphyxia [15]. Various evidence shows that low birth weight is a significant contributor to neonatal mortality, and that neonatal mortality is more likely to occur in newborns weighing < 2500 grams [33, 34]. Regarding this, studies conducted in Ethiopia [15], Tanzania [35] and Nigeria [36] also shown that newborn mortality was higher among infants with low birth weight.

Newborns with seizures had a 1.52 times higher risk of death compared to neonates who did not experience seizures. This might be due to the fact that convulsion causes the newborn to cease breathing, and if this interruption in breathing persists, it can lead to a drop in oxygen saturation, which threatens the life of the newborn [16]. Various studies conducted in Ethiopia, [15, 16], India [31, 37], and Tanzania [26] also showed similar findings.

Neonates that received resuscitation had a 2.11 times higher risk of death compared to their counterparts. Newborns who require immediate resuscitation following birth are usually hypoxic, with respiratory and metabolic acidosis. Furthermore, the difficulty of performing effective resuscitation combined with substandard technique may result in an inadequate clinical response [38]. Other findings also showed that neonatal mortality following resuscitation is high in low-income countries, with the risk of mortality highest in serious cases of the condition [38]. Moreover, the availability of priority equipment for neonatal resuscitation service is limited in Ethiopia, which would contribute to the significant association [39].

When compared to neonates admitted with mild asphyxia, newborns with severe and moderate asphyxia had an 11.55 and 3.5 times higher risk of mortality, respectively. Various scientific evidences also support this finding. The majority of deaths in neonates with asphyxia occur in severe and moderate cases due to brain damage, inadequate feeding, seizure, depressed myocardial function, and multi-organ dysfunction [17, 40–42]. Similar findings were also reported from studies conducted in Ethiopia [15, 17], rural Nigeria [22] and India [28, 31].

### Limitation of the study

Due to inconsistent registration and limited laboratory data, it was not possible to incorporate other variables that would affect the survival status of the neonates with asphyxia. Furthermore, the long-term complications among survivors were not addressed, and this finding would not be inferred to the general population.

## Conclusion

The magnitude of neonatal mortality among asphyxiated neonates in the study area was high. Admission weight, seizure, neonates who received resuscitation and stage of asphyxia were significantly associated variables with mortality among asphyxiated neonates. Therefore, special attention should be given to asphyxiated neonates with low admission weight and those who have had a seizure. Additionally, the timing, quality, and effectiveness of resuscitation might need further assessment and evaluation.

## Supporting information

S1 Dataset Stata dataset. (DTA)

## Abbreviations

AHR: Adjusted hazard risk
ANC: Antenatal care
APGAR: Appearance, Pulse, Grimace, Activity, Respiration
CI: Confidence interval
DSCH: Dessie Comprehensive Specialized Hospital
GA: Gestational age
MRN: Medical registration number
NICU: Neonatal intensive care unit
PROM: Premature rapture of membrane
